# Rapid Dilatation of the Cervix Uteri

**Published:** 1887-10

**Authors:** W. H. Wathen

**Affiliations:** Louisville, Kentucky


### *RAPID DILATATION OF THE CERVIX UTERI.*

By Dr. W. H. WATHEN, of Louisville, Kentucky.

The author had learned in the field of experience and observation of the bad results obtained in efforts to dilate the cervical canal by tents, or to enlarge or straighten it by incisions to cure dysmenorrhœa and sterility. He begged to call attention to the more satisfactory means of rapid dilatation with the bivalve or double-bladed dilators now in use, and especially to the substitution of an instrument of his device for Goodell's modification of Ellinger's dilator. If tents were used, he preferred the tupello to any other variety, it being less apt to cause septic inflammation than the sponge, and dilated more rapidly, regularly and better than the tangle. He referred to endometritis, pelvic hæmatocele, pelvic cellular or peritoneal inflammation, septicæmia, pyæmia and tetanus, as complications accompanying or following the use of tents, and did not believe that any good results, apparently, were permanently obtained. He claimed the two-bladed dilators are relatively aseptic, and are easily used, complete the operation at one sitting, and that the dilatation is comparatively free of immediate or subsequent dangers. It nearly always cures the dysmenorrhœa, and often removes the causes of sterility.

He thought the results of the incision of the cervix up to the vaginal junction, or through the os internal, anteriorly, posteriorly or bi-laterally, even more unsatisfactory than those following the use of tents. He dilates the cervix in his office, without a local or general anæsthetic, to the extent of half an inch, and allows the woman to walk or ride home a few minutes after. In dilatations of from three-quarters to one inch, he gives a hypodermic of morphia and atropia, then brings the patient under the influence of chloroform before operating. He urges great cleanliness, and all means to prevent septic infection. He uses three sizes of dilators; the largest the one he devised. He explained the points of superiority over Goodell's.

In conclusion, he urged that the operation should not be performed if there is any pelvic inflammation or trouble in the tubes or ovaries; and never, in any case, until we are reasonably positive that the cause of the trouble is in the cervical canal.

Dr. A. Martin, of Berlin, said that the dilatation of the uterus as an operation had undergone remarkable changes since its origination. The instruments shown by Dr. Wathen are an improvement. The great object is to open up the internal os. The degree of dilatation may be required to be less in some cases of small cervix. It is a question whether we have not been dilating in too many cases. I would not recommend that we go back to laminaria and sponge tents.

Dr. S. H. Weeks, of Portland, Me., claimed that every case of dysmenorrhœa was not due to mechanical obstruction of the cervix. The ovaries and tubes have much to do with it, and in his experience have been oftener at fault than the cervical canal. He was surprised at the freedom from danger reported in this operation. Professor Goodell has reported 100 cases with no bad result. These instruments are not devoid of danger, and bad results may come.

Dr. C. R. Reed, of Middleport, O., thought it quite puzzling to the young gynecologist to tell what to do, the testimony being so conflicting. Some authors reported that there never had been any bad results in the few hundred cases that they had operated on; others had frequent fatalities. I have more hesitancy in operating now than formerly. We cannot predict what the result will be in a given case.

Dr. Goelet, of New York, thought that the term rapid dilatation given to the operation was a misnomer. It had come into use to distinguish it from the dilatation by sponge or laminaria tents.

Dr. Steason, of North Carolina, performed dilatation fifty or seventy-five times a year for the past ten years, and keeps his patients in bed but two days. He has never had any fever, and has not used any antiseptic, except soap and water. He uses a glass rod to keep the canal open much the same as does Dr. Goelet, leaving it there sometimes as long as a week. He did not believe in the superfluous use of so much antiseptic.

Dr. A. Reeves Jackson, of Chicago, objected to the operation, because it did not do any good—at least permanent good.

Dr. Hadley, of Australia, never uses sponges and laminaria in dilatation, which he reviewed *in extenso.* He was in favor of the old treatment.

Dr. Harrf, of Ohio, recommended an original plan of his own, viz., the use of cornstalk pith. He entered his protest against rapid and extensive dilatation.

Dr. Lawrence, of Bristol, England, had used sounds constantly for years. His patients are constantly coming back. In the unimpregnated uterus, with proper precautions, this treatment can be used, but must not be left in too long. I use gelatin-coated sponge-tents, previously saturated with carbolic acid.

Dr. Wathen, in conclusion as to the question concerning his operating at his office, did it without any concern, and no anæsthetic. As to Emmett's opinion, we will concede a great deal to him in gynecology, but I do not see how he can be taken as an authority in this subject, seeing that he has not performed the operation, condemns it *in toto*, and will have nothing to do with it. The happy medium in this, as in other cases, is the thing to follow. No one would dilate for dysmenorrhœa caused by cellulitis or inflammation. The operation can be performed to straighten the neck of the uterus, and we can shorten the neck of the uterus by dilating the cervix and without amputation.

